# Successful management of refractory lymphoma-associated chylothorax with CT-guided direct embolization and pleurodesis

**DOI:** 10.1186/s42155-026-00780-x

**Published:** 2026-09-30

**Authors:** Yosuke Yamamoto, Jitsuro Tsukada, Masashi Tamura, Hiroki Yoshikawa, Yuji Furukawa, Ryo Ogawa, Mizuki So, Mamina Miyabayashi, Junya Tsuzaki, Seishi Nakatsuka, Yu Okubo, Akihisa Sakurada, Keisuke Asakura, Masahiro Jinzaki, Masanori Inoue

**Affiliations:** 1 Department of Radiology, Keio University School of Medicine, Tokyo, Japan; 2 Department of Radiology, Tokyo Saiseikai Central Hospital, Tokyo, Japan; 3 Department of General Thoracic Surgery, Keio University School of Medicine, Tokyo, Japan; 4 Department of Radiology, Fujita Health University, Aichi, Japan

**Keywords:** Refractory chylothorax, Malignant lymphoma, CT-guided intervention, Direct embolization, Thoracic duct embolization, Pleurodesis

## Abstract

**Background:**

Lymphoma-associated chylothorax is a challenging condition in which thoracic duct embolization (TDE) has limited efficacy because lymphatic leakage is often multifocal and difficult to localize. Furthermore, TDE performed without confirmed localization of the leakage site may increase upstream intralymphatic pressure and paradoxically worsen chylous leakage. Accurate localization of the leakage site before intervention is therefore essential.

**Case presentation:**

A 53-year-old man with low-grade B-cell lymphoma developed refractory bilateral chylothorax during chemotherapy. Conventional intranodal lymphangiography (INL) performed on two occasions failed to identify the leakage site, yet TDE was performed at the referring institution and was followed by marked clinical deterioration. Dynamic contrast-enhanced MR lymphangiography (DCE-MRL) subsequently demonstrated active lymphatic leakage from the residual lymphoma lesion. CT-guided direct embolization with n-butyl-2-cyanoacrylate (NBCA) was performed in four sessions, followed by surgical pleurodesis for persistent right-sided chylothorax, resulting in durable resolution without recurrence during 29 months of follow-up.

**Conclusion:**

This case highlights the importance of avoiding TDE without confirmed localization of the leakage site in refractory lymphoma-associated chylothorax. When conventional lymphangiography fails to identify the leakage site, DCE-MRL may facilitate targeted intervention and improve treatment planning. CT-guided direct embolization may represent a useful therapeutic option in carefully selected patients.

## Introduction

Lymphoma-associated chylothorax remains difficult to manage because lymphatic leakage is often multifocal and difficult to localize. Although thoracic duct embolization (TDE) has gained attention as a minimally invasive therapy, its efficacy in lymphoma-associated chylothorax remains limited, likely reflecting diffuse lymphatic abnormalities, multiple potential leakage sites, and difficulty in identifying the precise leakage point [1–3]. Furthermore, TDE performed without clear localization of the leakage site may increase upstream intralymphatic pressure and paradoxically worsen chylous leakage. We report a rare case of refractory lymphoma-associated chylothorax in which conventional intranodal lymphangiography (INL) failed to identify the leakage site and TDE was followed by clinical deterioration. Subsequent dynamic contrast-enhanced MR lymphangiography (DCE-MRL) successfully localized the leakage site, enabling targeted CT-guided direct embolization followed by surgical pleurodesis, resulting in durable resolution of chylothorax.

## Case presentation

A 53-year-old man with low-grade B-cell lymphoma developed bilateral chylothorax during the second cycle of bendamustine chemotherapy. Although chemotherapy reduced the tumor burden, right-sided chylothorax persisted with a daily output of approximately 1 L despite conservative management, including dietary modification, total parenteral nutrition, and octreotide. INL performed at the referring institution failed to identify the leakage site. Nevertheless, retrograde transvenous TDE with n-butyl-2-cyanoacrylate (NBCA) was performed via the left venous angle. Following TDE, bilateral chylothorax markedly worsened, requiring bilateral chest drainage, and the patient was referred to our institution.

**Fig. 1:**
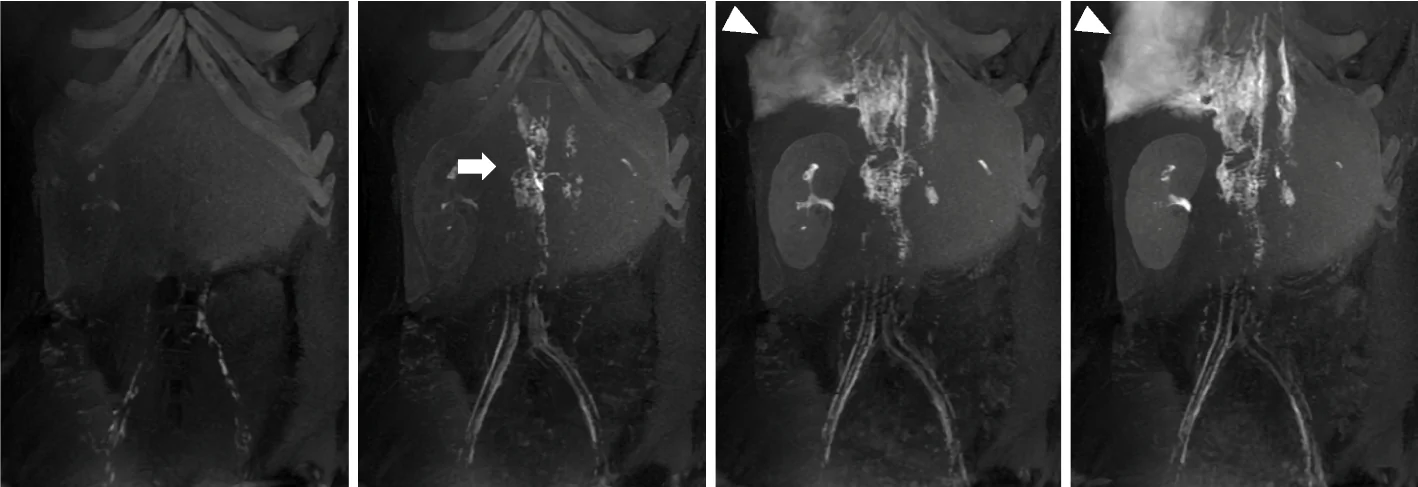
Dynamic contrast-enhanced MR lymphangiography (DCE-MRL) demonstrating time-resolved lymphatic leakage. Serial coronal maximum intensity projection (MIP) images show the dynamic flow of gadolinium-based contrast through the central lymphatic system. Initial frames depict contrast accumulation in the lumbar and para-aortic lymphatic vessels (arrow). In the later frames, extensive leakage is visualized into the right pleural cavity (arrow heads) from the region of the residual lymphoma lesion, confirming the site of chyle extravasation

Repeat INL again failed to identify a clear leakage site. DCE-MRL subsequently demonstrated active lymphatic leakage from the scarred lesion of the regressed lymphoma into the pleural cavity (Fig. 1). Based on these findings, CT-guided direct embolization was performed in four sessions over 2 months. After CT-guided needle placement, a small amount of iodinated non-ionic contrast medium was injected to visualize the abnormal lymphatic channels within the lesion.

**Fig. 2:**
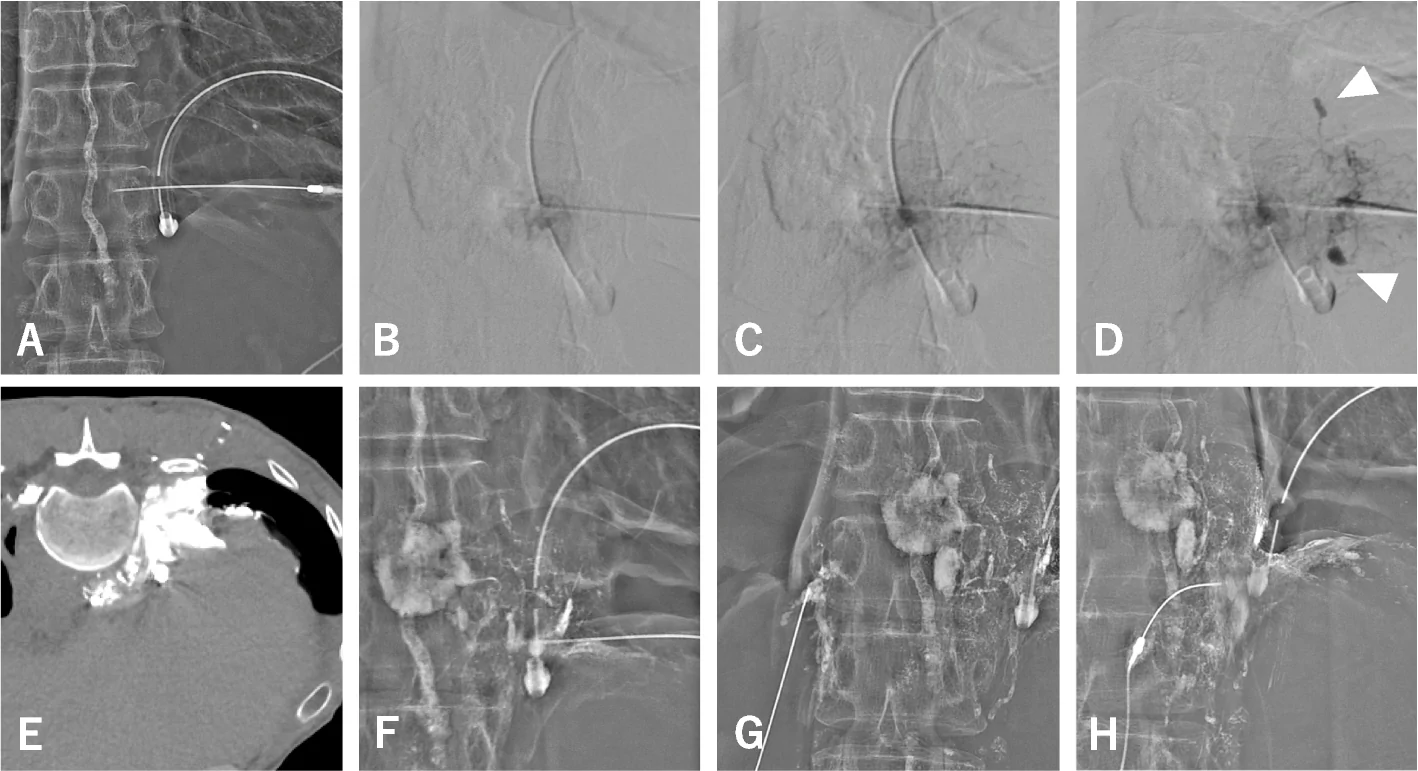
Stepwise CT-guided direct embolization of a residual lymphoma lesion with NBCA in a patient with refractory chylothorax. **A**–**D** First embolization session: Fluoroscopic images showing progressive injection of n-butyl-2-cyanoacrylate (NBCA) mixed with iodized oil (1:2 ratio) through a 21-gauge needle directly inserted into the residual lymphoma lesion under CT guidance. The glue cast gradually outlines the lymphatic channels suspected to be the source of chyle leakage (arrow heads). **E** Non-contrast axial CT image obtained immediately after the first embolization, showing the distribution of the glue cast within the targeted lesion. **F**–**H** X-ray images from the second to fourth embolization sessions performed over a two-month period, showing repeated NBCA injections and progressive coverage of the suspected lymphatic leak points

**Fig. 3:**
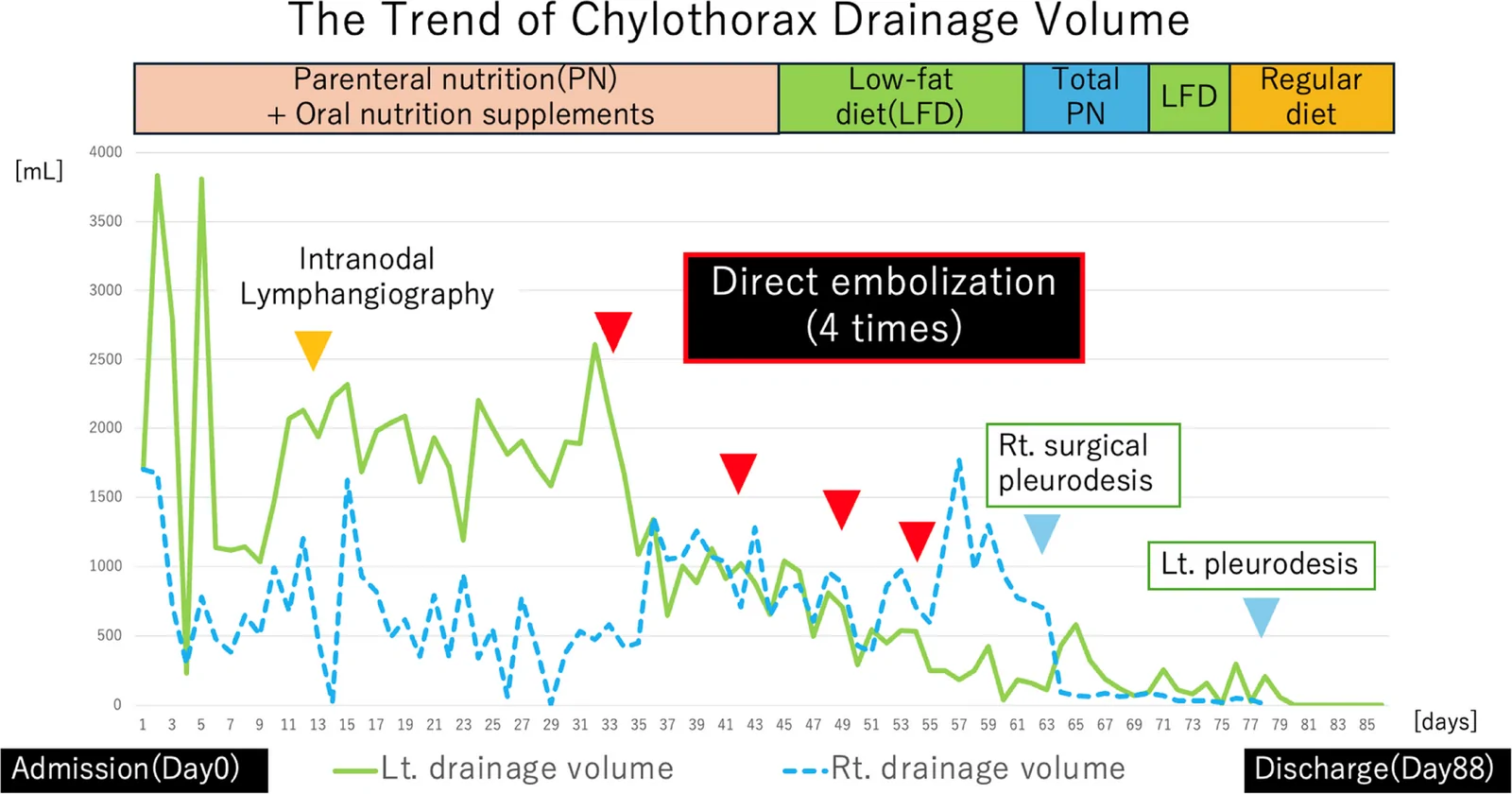
Treatment course of refractory bilateral chylothorax secondary to malignant lymphoma. This figure illustrates the time course of pleural drainage volumes from the left (solid green line) and right (dashed blue line) thoracic cavities. Initially, conservative management was initiated using parenteral nutrition and oral nutritional supplements (PN + ONS), followed by alternating periods of low-fat diet (LFD) and PN, and eventual transition to a regular diet (RD). On day 12 (orange arrow), intranodal lymphangiography was performed, but no obvious leakage site was identified. Between days 35 and 55, CT-guided direct embolization was performed four times (red arrowheads), resulting in a marked decrease in drainage volume, especially on the left side. Due to persistent right-sided chylothorax, surgical pleurodesis via thoracotomy was performed on day 61 (purple arrow), and additional talc pleurodesis was conducted on the left side on day 75 (purple arrow). Both chest drains were subsequently removed, and the patient was discharged on day 88

Once these channels were opacified, regardless of whether contrast extravasation into the pleural cavity was observed, NBCA mixed with iodized oil at a 1:2 ratio was immediately injected (Fig. 2). Pleural drainage was markedly reduced, particularly on the left side. Persistent right-sided chylothorax ultimately required surgical pleurodesis. Combined percutaneous embolization and surgical treatment achieved durable resolution without recurrence during 29 months of follow-up (Fig. 3).

## Discussion

TDE is an established treatment for refractory chylothorax, with reported clinical success rates approaching 80% in technically successful cases [4]. However, outcomes are considerably less favorable in lymphoma-associated chylothorax, where previous reports have described the need for repeated lymphatic interventions or substantially lower success rates [2, 3]. This likely reflects diffuse lymphatic abnormalities, multiple potential leakage sites, and difficulty in accurately localizing the leakage site before intervention [2, 3, 5]. Recent multidisciplinary reviews have therefore emphasized that management should be individualized using combinations of conservative, interventional, and surgical approaches [5]. In our case, TDE was performed despite unsuccessful INL and was followed by marked worsening of bilateral chylothorax. Although causality cannot be established from a single case, thoracic duct occlusion may have increased upstream intralymphatic pressure and exacerbated pre-existing lymphatic leakage. Maruyama et al. recently demonstrated that thoracic duct outflow obstruction increases thoracic duct pressure above central venous pressure, creating a state of lymphatic hypertension [6]. In our patient, because the leakage site had not been identified before TDE, thoracic duct occlusion may have increased pressure within disrupted lymphatic channels surrounding the residual lymphoma lesion, thereby aggravating lymphatic leakage into the pleural cavity. These experimental findings provide physiological support for the mechanism underlying the clinical deterioration observed in our patient.

Conventional lymphangiography twice failed to detect the leakage site, whereas DCE-MRL clearly demonstrated active leakage from the regressed lymphoma lesion. Because DCE-MRL uses gadolinium-based water-soluble contrast, it can depict finer lymphatic structures than conventional Lipiodol lymphangiography, thereby improving visualization of subtle or multifocal leakage [7, 8].

The deterioration following non-targeted TDE fundamentally changed our therapeutic strategy. Rather than pursuing further empirical lymphatic intervention, we first prioritized precise localization of the leakage site using DCE-MRL. Identification of the residual lymphoma lesion as the source of leakage enabled targeted CT-guided embolization, which substantially reduced lymphatic drainage and stabilized the patient's condition sufficiently to allow definitive surgical pleurodesis. Pre-procedural DCE-MRL demonstrated bilateral pleural leakage, although the leakage was more prominent on the right side. We speculate that the left-sided leakage was less extensive and therefore effectively controlled by embolization alone, whereas the right side likely involved more extensive or multiple disrupted lymphatic channels that could not be completely embolized. Successful control of the left-sided leakage also made surgical pleurodesis a feasible treatment option, whereas bilateral leakage had initially precluded definitive surgical management. Earlier localization of the leakage site by DCE-MRL before TDE might have enabled targeted intervention as the initial treatment strategy. This approach might have avoided non-targeted thoracic duct occlusion, prevented clinical deterioration, and reduced the need for subsequent surgical intervention. Direct puncture embolization of lymphomatous lesions has rarely been reported [9]. In our case, injection of the NBCA mixture demonstrated contrast extravasation from the lesion into the pleural cavity, confirming that the residual lymphoma lesion was the source of lymphatic leakage. CT-guided direct embolization substantially reduced lymphatic drainage and stabilized the patient's condition sufficiently to permit definitive surgical pleurodesis.

This case highlights the importance of avoiding TDE without confirmed localization of the lymphatic leakage site in refractory lymphoma-associated chylothorax. When conventional lymphangiography fails to identify the leakage site, DCE-MRL should be considered before TDE to guide individualized image-guided intervention. CT-guided direct embolization may represent a valuable therapeutic option in carefully selected patients with refractory lymphoma-associated chylothorax.

## Data Availability

All data generated or analyzed during this study are included in this published article. Additional details are available from the corresponding author upon reasonable request.

## Abbreviations

DCE-MRL: Dynamic contrast-enhanced magnetic resonance lymphangiography
INL: Intranodal lymphangiography
NBCA: N-butyl-2-cyanoacrylate
TDE: Thoracic duct embolization
